# Optimization of Multilayer Films Composed of Chitosan and Low-Methoxy Amidated Pectin as Multifunctional Biomaterials for Drug Delivery

**DOI:** 10.3390/ijms23158092

**Published:** 2022-07-22

**Authors:** Joanna Potaś, Agnieszka Zofia Wilczewska, Paweł Misiak, Anna Basa, Katarzyna Winnicka

**Affiliations:** 1 Department of Pharmaceutical Technology, Faculty of Pharmacy, Medical University of Białystok, Mickiewicza 2C, 15-222 Białystok, Poland; joanna.potas@umb.edu.pl; 2 Department of Polymers and Organic Synthesis, Faculty of Chemistry, University of Białystok, Ciołkowskiego 1K, 15-245 Białystok, Poland; agawilcz@uwb.edu.pl (A.Z.W.); p.misiak@uwb.edu.pl (P.M.); 3 Department of Physical Chemistry, Faculty of Chemistry, University of Białystok, Ciołkowskiego 1K, 15-245 Białystok, Poland; abasa@uwb.edu.pl

**Keywords:** polyelectrolyte complex, polyelectrolyte multilayer, chitosan, pectin, buccal drug delivery materials

## Abstract

Polyelectrolyte multilayers (PEMs) based on polyelectrolyte complex (PEC) structures are recognized as interesting materials for manufacturing functionalized coatings or drug delivery platforms. Difficulties in homogeneous PEC system development generated the idea of chitosan (CS)/low-methoxy amidated pectin (LM PC) multilayer film optimization with regard to the selected variables: the polymer ratio, PC type, and order of polymer mixing. Films were formulated by solvent casting method and then tested to characterize CS/LM PC PECs, using thermal analysis, Fourier transform infrared spectroscopy (FTIR), turbidity, and zeta potential measurements. The internal structure of the films was visualized by using scanning electron microscopy. Analysis of the mechanical and swelling properties enabled us to select the most promising formulations with high uniformity and mechanical strength. Films with confirmed multilayer architecture were indicated as a promising material for the multifunctional systems development for buccal drug delivery. They were also characterized by improved thermal stability as compared to the single polymers and their physical mixtures, most probably as a result of the CS–LM PC interactions. This also might indicate the potential protective effect on the active substances being incorporated in the PEC-based films.

## 1. Introduction

Materials based on polyelectrolyte complexes (polycomplexes, PECs) have been under detailed investigation over the last years as structures with great possibilities in multifunctional systems development. Multilayer PEC-based films are characterized by huge potential in tissue engineering and chemical or pharmaceutical technology. They are characterized by unique properties arising from combining polymers with different characteristics enriched with the special properties of PEC structures [1,2,3]. Preparation of PECs does not require any addition of toxic cross-linking agents but usually occurs after mixing aqueous solutions of polyelectrolytes. Since rapid interactions between oppositely charged polymers might lead to PECs’ coacervation/precipitation, separation of insoluble PEC particles is one of the methods to preserve the homogeneity of the systems with regard to their further utilization [4]. Considering the number of factors that usually impact PECs’ performance, such as polymer properties (molecular weight, charge distribution, pKa, solubility, chain flexibility), ionic strength, pH values affecting the polyelectrolytes’ ionization degree, temperature, concentration of the interacting compounds, polymer ratio, and preparation technique, precise evaluation of the polyelectrolytes should always be performed prior to the highly controlled process of PEC formation [5,6,7,8]. There are numerous criteria of PEC classification; nevertheless, differentiation of polycomplexes on water-soluble non-stoichiometric and water-insoluble stoichiometric structures is most often invoked [5].

Polyelectrolyte multilayers (PEMs), composed of alternately deposited layers of anionic and cationic polyions separated with an interfacial layer of ionically interacting polymeric chains creating PECs, are recognized as particularly interesting materials for obtaining functionalized coatings and drug delivery platforms [1]. While polycomplex-based systems obtained by mixing the polymers with opposite charges might be treated as systems with low homogeneity as a result of easily precipitating PEC particles [4,5], the technique of gradual deposition of polyelectrolyte solutions might enable one to prepare uniform composites with usually well-preserved physicochemical performance of the individual components. It is particularly valid for those composites for which maintaining the original behavior of polymers is necessary for the multifunctional character of PEMs.

Among the plethora of polymers being utilized for PEC-based systems technology, chitosan (CS)—a linear polycation with positively charged amino groups [9]—has been widely investigated. The presence of amino groups is mainly responsible for the unique characteristics of CS, including mucoadhesive [10], antimicrobial [11], and anti-inflammatory or hemostatic properties [12]. The possibility of developing multifunctional CS-based materials with therapeutic effects supported by the polycation by itself was the main reason for designing buccal carriers for clotrimazole delivery. Pros and cons of high-molecular-weight CS and high-methoxy amidated pectin (HM PC) multilayer composites based on interpolymer complexation of the oppositely charged polymer chains showed the need for careful adjustment of the technological procedure of the films since many different factors turned out to shape the final PEC-based product. PC is, similarly to CS, a natural origin polysaccharide with an essentially linear structure composed of D-galacturonic acid units linked by α-(1, 4)-glycosidic bonds [13]. According to the degree of methyl esterification, high-methoxy (HM PC) (≥50%) and low-methoxy amidated PC (LM PC) (<50%) can be distinguished. While HM PC jellifies in the presence of soluble solids at a pH around 3, LM PC requires calcium or other divalent ions for a gelation process. The mechanism of LM PC gelation strictly corresponds to the “egg–box” model, where calcium ions are ionically bound to carboxylic groups of galacturonan monomers [14]. Calcium reactivity is a parameter that describes the amount of calcium required for the highest PC gel strength at a certain concentration of soluble solids. Upon increasing the calcium content, PC gel becomes firmer up to a certain ion concentration value, beyond which the mechanical strength noticeably declines. PC is treated as an interesting candidate for pharmaceutical use [15,16,17], including for the technology of the films [18,19,20,21]. As an anionic biopolymer with carboxylic groups capable of ionic interactions with positively charged polyelectrolytes, PC has been under careful investigation for PECs formation [1]. The mucoadhesive properties of LM PC have shown its great potential for mucosal drug administration [16]. The development of CS/LM PC multilayers can be regarded as particularly beneficial for the improved biocompatibility of CS as well as the increased mechanical strength of the hybrid PEC materials as compared to the single-polymer-based systems [17,22]. Layer-by-layer deposition of the polyelectrolyte solutions using the solvent evaporation technique aimed to eliminate the risk of polycomplexes’ precipitation since preliminary studies pointed out the high susceptibility of CS/PC blends to phase separation.

Taking into consideration the increasing potential of polycomplex materials with CS as novel and complex drug delivery platforms and our experience in PEC-based systems fabrication [23,24], the idea was to dive deeper into technological aspects of the interpolymer complexation process. Furthermore, the aim was to verify whether the selection of the internal and external stimuli that might potentially influence the films’ characteristics can help develop optimized CS/PC materials. For this purpose, medium-molecular-weight CS and low-methoxy amidated PC with different calcium reactivity were utilized. In the framework of PEMs optimization, different PC types, polymer ratios, and orders of polyelectrolyte mixing were applied. The prepared films were analyzed with regard to CS/LM PC PECs’ characterization. Thermal analysis, Fourier transform infrared spectroscopy (FTIR), turbidity, and zeta potential measurements were performed. The internal structure of the films was visualized by scanning electron microscopy (SEM), although visual observations were equally important since the films were morphologically diversified. Mechanical and swelling performance of the composites were also evaluated and became a valuable source of information about CS/LM PC behavior against the factors being modulated. Despite the attention given to the pharmaceutical assessment of PEMs, the paper focuses on searching for correlations between the intensity of ionic CS–PC interactions and the films’ performance. The question is whether careful optimization of the interpolymer complexation process might be helpful in the development of highly functionalized PEMs, with potential application for buccal drug administration being the object of our special interests.

The increasing problem of oromucosal infections necessitates development of buccal products resistant to the eroding capacity of saliva. From the other side, the buccal mucosa might be treated as an alternative route of systemic drugs administration. Considering the specificity of the oral cavity, PEM development and optimization aims to obtain non-toxic, biodegradable films with satisfactory mechanical strength and retentivity. The structure of the systems composed of polymers with different behaviors upon contact with body fluids creates an opportunity for controllable release of a drug/drugs at the application site [25]. Selection of CS and LM PC—safe, mucoadhesive polymers particularly suited to mucosal administration, but with varied water-solubility and sensitivity to ions—can be recognized as highly beneficial for developing buccal films. It is expected that maintained physical integrity of the films in contact with saliva will enable prolonged delivery of the active substance as a consequence of the stable, slowly disintegrating structure of PEC, modulated by gradually swelling and eroding PC macromolecules.

## 2. Results and Discussion

### 2.1. Mechanical Properties

The mechanical strength of the films is important for their physical integrity during both technological and application procedures. Parameters such as tensile strength (σ_s_), elongation at break (ε_s_), Young’s modulus (E), or tear resistance (TR) might be helpful in the evaluation process of the films. The σ_s_ and TR describe the stress needed for breaking of a film, and ε_s_ corresponds to the possible material deformation until it tears. E measures the susceptibility of a film to being deformed and might be indicated by the slope on the stress/strain curve drawn by Texture Analyzer [26,27]. In this study, great attention was paid to the mechanical analysis since visual differences in the physical performance of the formulations F1–F16 have been observed. Evaluation of the mechanical properties was regarded as crucial for the optimization of the buccal films. Satisfactory elasticity and strength of dosage forms are important with regard to both patient comfort and resistance to the saliva’s eroding capacity when applied to the buccal mucosa [26].

**Figure 1:**
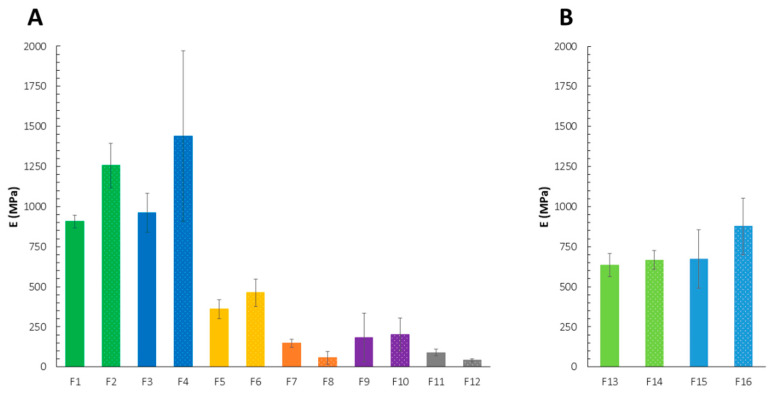
Mechanical properties of the films F1–F12 (**A**) and F13–F16 (**B**) expressed as Young’s modulus (E) (mean ± standard deviation (SD), *n* ≥ 3, *p* < 0.05). Formulations with the same polymer ratio and deposition order, but different LM PC type were indicated with the same color.

The highest values of E were calculated for the films with an equal amount of CS and PC (F1–F4) (Figure 1, Table 1). To recognize potential differences resulting from either various polymer concentrations or the intensity of PECs formation at each polymer ratio, the mechanical behavior of F13–F16 was also evaluated. Despite different total polymer content, E values were comparable for both F1–F4 and F13–F16, especially for these formulations with the PC layer casted as the first one. Simultaneously, poorly repeatable measurements were noted for the films with the PC layer deposited first (F4), which might have arisen from the visually noticeable heterogeneity of these formulations. It might be then assumed that this order of polymer casting promoted the complexation of CS’s amine groups with carboxylic groups of the polyanion. The films F5–F8—being mainly composed of PC—showed an inhomogeneous appearance after drying, probably as a result of less or more severe interactions between PC and easily penetrating CS chains. In fact, noticeable thickening and folds with air bubbles in the structure of the multilayers were observed for the F5–F8 formulations. The films with the ratio of CS to PC 10:1 were relatively uniform in color and transparency regardless of the polyelectrolyte mixing order. When it comes to the mechanical performance of the non-stoichiometric films, harder and more brittle formulations were obtained when CS was deposited as the first layer. On contrary, for the F1–F4 and F13–F16 films, the values of E were similar despite the preparation techniques used.

**Figure 2:**
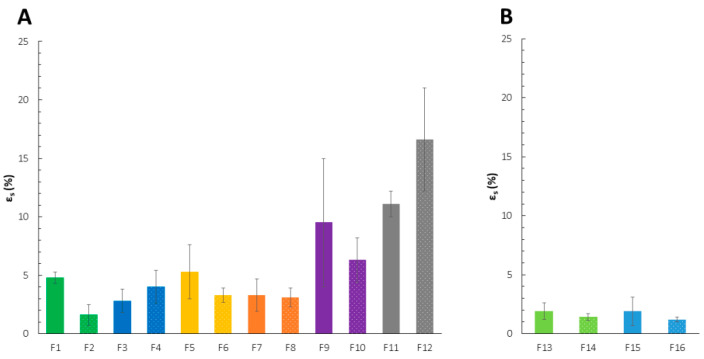
Mechanical properties of the films F1–F12 (**A**) and F13–F16 (**B**) expressed as elongation at break (ε_s_) (mean ± SD, *n* ≥ 3, *p* < 0.05). Formulations with the same polymer ratio and deposition order, but different LM PC type were indicated with the same color.

Among all tested films, those with higher content of CS (F9–F12) showed higher values of ε_s_, indicating outstanding elasticity compared to the others (Figure 2). However, due to the high standard deviation values, the main factors responsible for such mechanical behavior cannot be indicated. Only for films with PC 020 (F10, F12), the deposition order affected the values of ε_s_—the films with PC deposited as the first one were more flexible compared to those with preliminary casted CS. The parameter values were comparable to the physicomechanical characteristics of the vaginal blend CS/PC films developed by Mishra et al. [28]; nevertheless, the great elasticity of these formulations was probably determined by the very high (30% (*v*/*v*)) concentration of glycerol as a plasticizer. Taking into consideration the previous observations on the correlation between the PECs formation process and the fragility of the films, the superiority of CS over PC content in F9–F12 might result in less intense polycation–polyanion interactions. This was confirmed by the significantly enhanced values of ε_s_ and more flexible films formation. It might be concluded that low participation of carboxylic groups in CS complexation resulting from both low PC content and relatively weak ionic strength of the polyanion affected the mechanical performance, being mostly “shaped” by free, not complexed, CS. Drying time can also influence the performance of the films. More pronounced water evaporation from the external CS layer—being exposed to a temperature for a longer time—resulted in lower elasticity. The impact of PEC structures on the mechanical behavior of polycation/polyanion materials was presented by Tejada’s group on high-molecular-weight CS and high-methoxy amidated PC films as potential drug carriers for an antifungal miconazole nitrate [29]. Blend films consisting of a mixture of 3% (*w*/*v*) CS and 3% (*w*/*v*) PC solutions were more resistant to mechanical deformation as both the increased σ_s_ and reduced ε_s_ values suggested. The physicomechanical performance of PEC-based films highly corresponds to the polyelectrolytes’ characteristics, especially their molecular weight [29,30,31].

It has been clearly shown that the polymer content is not the only factor determining the mechanical strength of the films. The degree of interpolymer complexation is equally important since formulations with the same total amount of polyelectrolytes but various polymer ratios performed differently in the mechanical test. The composites F13–F16 with a high probability of stoichiometric PECs formation were noticeably more resistant to mechanical pressure compared to the delicate and more elastic F5–F12—films with low participation of electrostatically interacting amine–carboxylic groups, which is important for the enhanced strength of the films. As already mentioned, the impact of PEC particles on the development of more brittle and hard composites is well known in the literature [31,32].

### 2.2. Films Morphology

**Figure 3:**
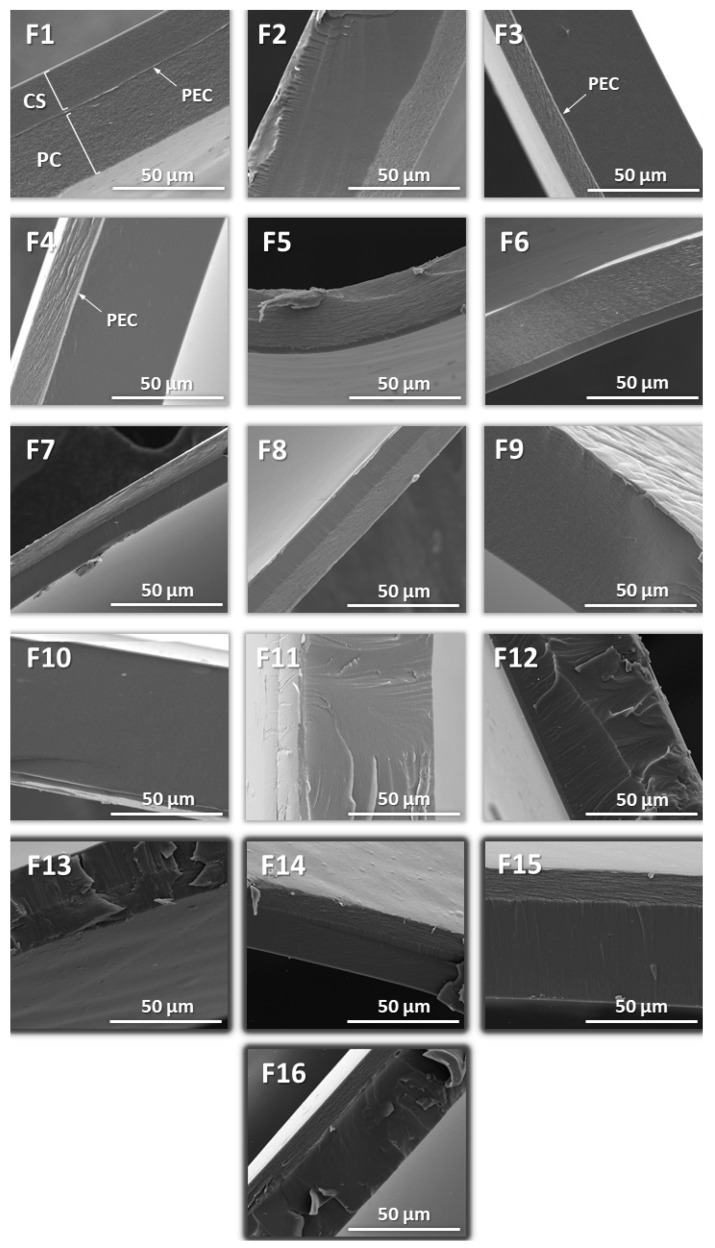
Representative SEM images of F1–F16 films under 3000× magnification.

**Figure 4:**
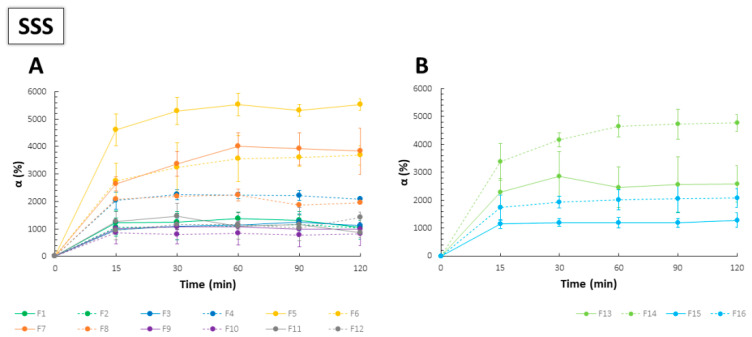
Swelling behavior of F1–F12 (**A**) and F13–F16 (**B**) films in the simulated saliva solution (SSS) (mean ± SD, *n* = 3).

SEM analysis showed the layered structure of the films, composed of the relatively homogeneous and dense layer of CS and a noticeably less packed PC layer with visible delamination signs (Figure 3). Ionically interacting polymer chains were recognized as a thin, brighter area at the site of PC–CS contact; however, this was not observed for all analyzed formulations. Compared to F1 and F13, which consisted of two layers with similar thicknesses, F2–F4 showed an unevenly divided layered structure despite identical polymer content in both main layers of CS and PC. Cross-sections of F13 turned out to be full of cracks, with areas of inhomogeneously placed PECs particles. The PC-oriented layer of CS was illustrated for F15–F16. The horizontally directed structure of PC resulted in the formation of a significantly thinner polyanion layer compared to CS [13]. It was noticed that in the case of the composites with the PC layer distributed as the second one (F1–F2 and F13–F14), use of PC 010 increased the thickness and elasticity of the films. With comparison to F9–F12 characterized by the ratio of CS to PC 10:1 (*w*/*w*), films with dominant content of PC (CS to PC 1:10, *w*/*w*) were noticeably thinner, probably as a result of the linear structure of PC. The F3–F4 films were characterized visually as very delicate films with low elasticity, which might be a result of the presence of a relatively thick interlayer of PEC, as shown in Figure 4. Regardless of PC type, deposition of CS on a pre-jellified PC layer resulted in intense interpolymer complexation with a visible layer of CS/PC PEC. This is highly correlated with the results obtained in the mechanical analysis (Section 3.1), although it cannot be compared with the performance of the CS/sodium hyaluronate films developed by Kononova et al. [33]. The fact that deposition of an anionic sodium hyaluronate on the CS surface resulted in more ordered and denser PEC interlayer formation compared to the reverse addition order might indicate the individual and unique character of each polycomplex structure depending inter alia on the polyanion used.

According to the investigations made by Schlenoff’s and Fares’s groups [34,35], the importance of the site diffusion mechanism in the formation of the multilayers should be highlighted at this point. Rather than relying on the interdiffusion of polyions molecules throughout an entire film in PEMs fabrication, the aforementioned research groups pointed out the role of changes within its extrinsic sites composed of polyelectrolytes monomers balanced by counterions. The analysis of F5–F8 films showed very interesting results with regard to the potential mechanism of PEMs’ formation. While the distribution of PC on firstly deposed CS led to thin film formation with, as anticipated, the dominant layer of PC being the result of a ten-fold larger amount of the polyanion versus polycation (F5–F6), the reverse order of polymer mixing led to almost equally divided bilayer composites (F7–F8). We suspect that strong electrostatic interactions between PC and CS added in the second step favored tight loading of linear chains of PC according to the polymer-dominated diffusion mechanism [34,35].

The order of polyelectrolyte deposition turned out to be crucial for the intensity of CS–PC ionic interactions. As the SEM images showed (Figure 3), reorganization of both CS and PC chains toward the polymer contact surface was observed; nevertheless, it was mainly characteristic for F8. The F7–F8 films were visually inhomogeneous, with the presence of wrinkles and areas of PEC precipitates placed throughout the whole films, which was also illustrated by the SEM analysis. The films F5–F6 had inhomogeneous cross-sections with a noticeably dominant layer of PC divided from a thin coat of CS with a visible PEC layer. The presence of air bubbles affected the non-uniform morphology of the films. Very interesting observations were made for F9–F12. These composites were homogeneous in the visual evaluation; nevertheless, deposition of PC of each type in the second step (F9–F10) resulted in the polyelectrolytes mixing across the whole width of the films and therefore the monolayer architecture. In the SEM analysis, a very dynamic process of 2% CS interpenetration throughout ten-fold less concentrated PC was illustrated.

### 2.3. Swelling Capacity

Considering the presence of calcium ions in different biological fluids [26], their interaction with PC chains leading to externally simulated gelation provides a tool for tailored and prolonged delivery of active substances at the application site. Although the susceptibility of the polyanion to other positive ions (mainly magnesium and potassium) cannot be omitted, calcium is particularly effective in PC gelation by forming bridges between carboxylic groups of two neighboring PC chains and divalent ions, which are additionally supported by hydrogen bonds.

The highest values of α in SSS were noted for the films with the ratio of CS to PC 1:10, which might have suggested the main role of PC in swelling and disintegration processes (Figure 4). Films F9–F12, being mainly composed of CS, showed significantly lower values of α, and no important differences were noted for both types of PC. Complexation of carboxylic groups of PC with amine groups of CS limited the capability of the polyanion for the SSS uptake, which can be regarded as characteristic for PECs-based materials. As stoichiometric PECs were known for their dense and rigid structure, a limited swelling process was repeatedly observed [32,36]. On the contrary, non-stoichiometric PECs, including those with PC as an anionic component, were mainly characterized by a high capacity of water uptake due to the formation of macroporous film structure [37,38]. While F1–F2 and F13–F14 showed varied swelling performance, mass changes upon contact with SSS noted for F3–F4 and F15–F16 were comparable (analogically prepared formulations with the same polymer ratio). For composites with predominant PC, more easily jellifying PC 020 hampered water entrance into the film matrix; nevertheless, the values of α were still markedly higher as compared to the other polymer ratios. Furthermore, films with the lowest values of thickness recorded by SEM (F5–F8 and F13–F14) showed great swelling capacity, which might be caused by the high porosity and “elasticity” of the PC layer despite the initially condensed linear structure of the polymer. In comparison to the swelling behavior of PEC-based films with high-molecular-weight CS and HM PC, which were the object of our previous studies [24], the use of LM PC resulted in obtaining relatively stable and slowly disintegrating films beneficial for prolonged drug delivery. In addition, buccal dosage forms are a group of products for which high retentivity is necessary due to the eroding properties of the saliva.

**Figure 5:**
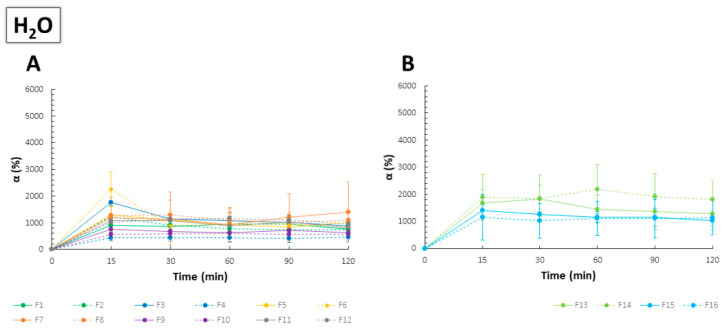
Swelling behavior of F1–F12 (**A**) and F13–F16 (**B**) films in purified water (mean ± SD, *n* = 3).

Except for F3–F4, no significant variations in water absorption were noted between the formulations with different PC type (Figure 5). The swelling performance of F1–F2 and F9–F12 were comparable regardless of the medium utilized, while the other composites were characterized by significantly weaker water entrance as compared to the SSS absorption. According to the above, it might be assumed that the high attendance of PC chains in the interpolymer complexation affected the limited ability of the polymer to jellify and easily swell the matrix formation.

### 2.4. Thermal Characteristics

**Figure 6:**
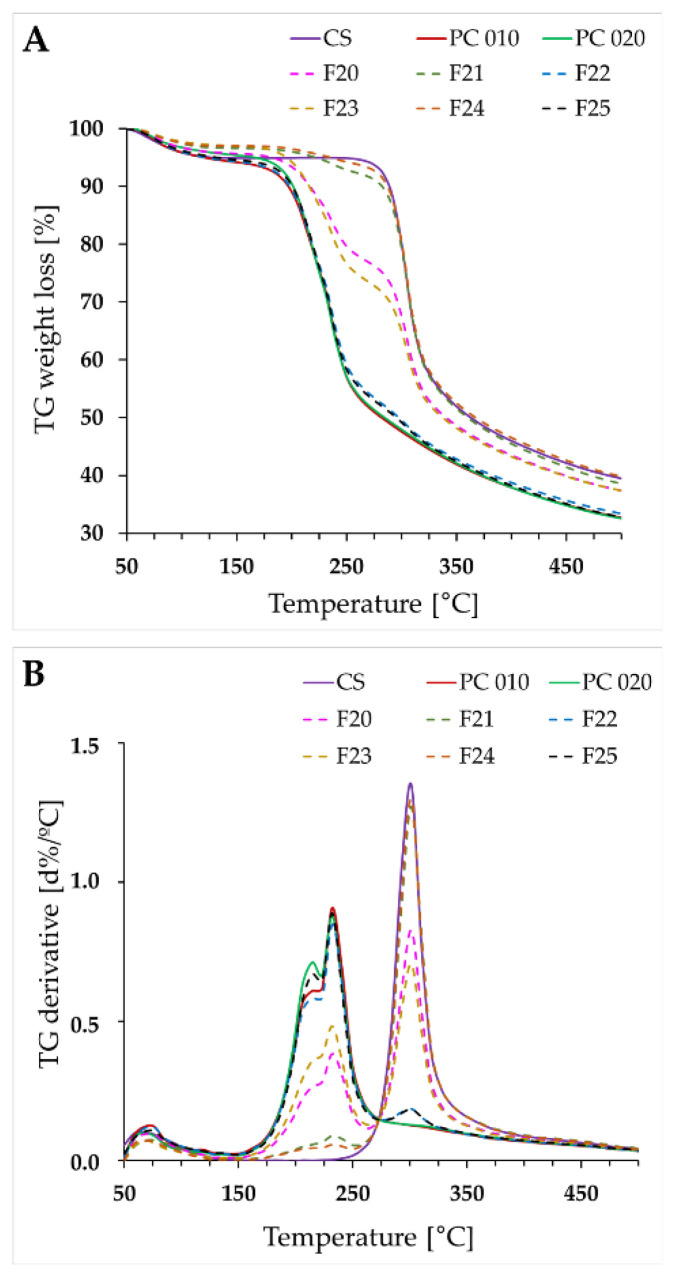
TGA profiles (**A**,**B**) of CS, PC 010, PC 020, and their physical mixtures in the polymer ratios corresponding to the films composition (F20—CS:PC 010 1:1, F21—CS:PC 010 10:1, F22—CS:PC 010 1:10, F23—CS:PC 020 1:1, F24—CS:PC 020 10:1, F25—CS:PC 020 1:10).

The weight loss was observed at the range of 50–150 °C for the “pure” polymers and their physical mixtures and was related to the elimination of moisture (Figure 6). Gradual degradation of the samples was recorded in the thermogravimetric analysis (TGA). The “pure” polyelectrolytes have undergone a two-step and three-step decomposition process for CS and both PCs, respectively. The first one was related to the water evaporation, with subsequent depolymerization of polymer chains [39]. For the polymer blends with the ratio of CS to PC 1:1, TG curves corresponded to the degradation processes characteristic of the single components. Simultaneously, thermograms of the physical mixtures with a predominance of either polycation or polyanion were very similar to those recorded for “pure” CS/PC.

**Figure 7:**
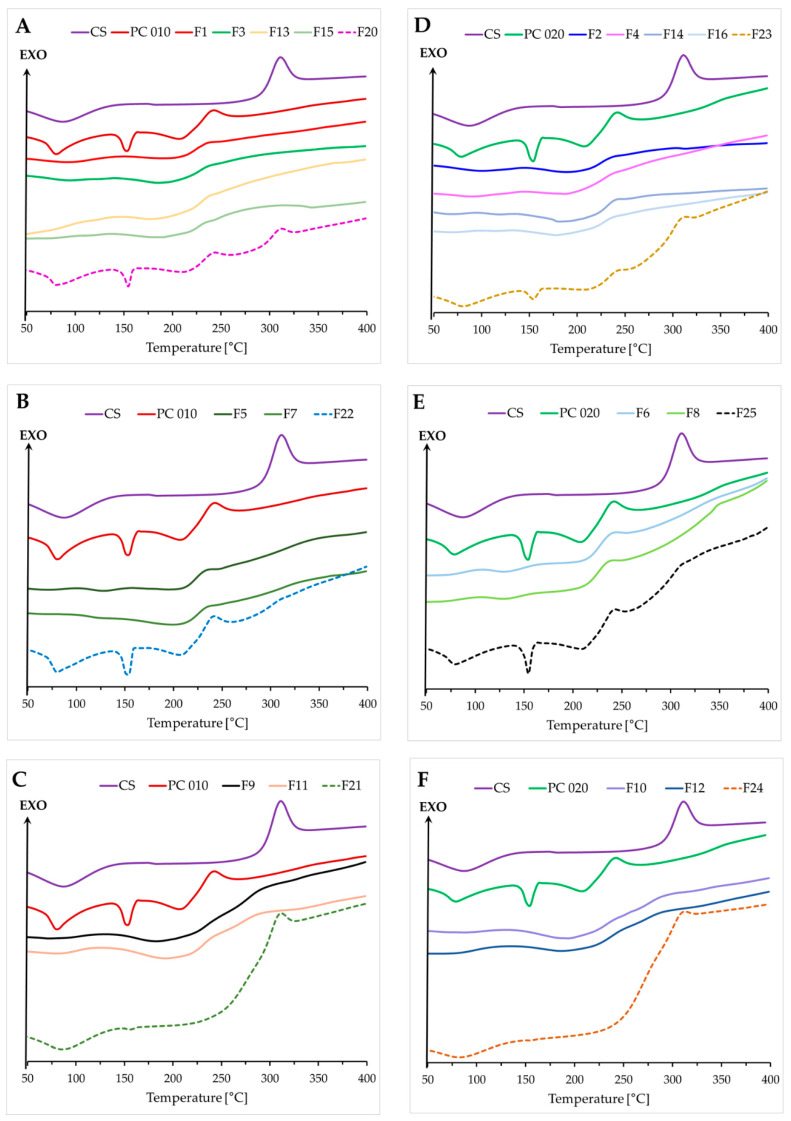
DSC curves for “pure” polyelectrolytes (CS, PC 010, PC 020), the films F1–F16, and the corresponding physical mixtures (F20–F25) (**A**–**F**).

Calorimetric curves for the physical mixtures (Figure 7, F20–F25) and “pure” compounds were consistent. Any differences were correlated with the varied polymer content. Differential scanning calorimetry (DSC) thermograms for single polymers and their physical mixtures presented wide endothermic peaks in the range of 50–120 °C, probably related to the evaporation of the absorbed moisture [40]. They were not observed for the developed films, probably as a result of the drying process. The preparation of the films led to the formation of products characterized by new thermal properties. Analysis of the pervaporation membranes consisting of CS and hyaluronic acid in the ratio of 1:1 (*w*/*w*) performed by Kononova’s research group also indicated the enhanced thermal stability of the PEC-based product [33]. No differences in the performance of both examined sides of the films were noted. The sharp endothermic peak at approximately 150 °C, probably corresponding to the melting point of PC 010/020, was absent for all analyzed films [15,16], indicating the possible transformation of PC into an amorphous form during the preparation of the films. The DSC curve recorded for CS was typical of an amorphous form of the compound [41]. The degradation process of both CS and PC was recorded above 200 °C; nevertheless, noticeably slower decomposition of the films was observed as compared to the “pure” polymers or their physical mixtures. This might be related to interpolymer bonding formation.

### 2.5. Turbidity

**Figure 8:**
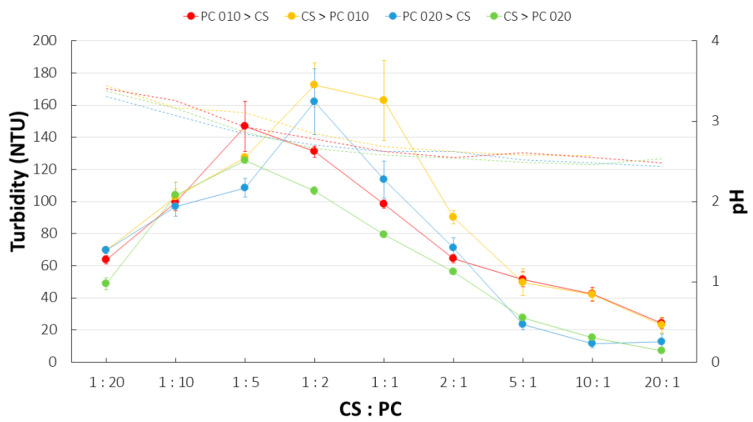
Turbidity (continuous line) and pH fluctuations (dashed line) recorded for CS/PC mixtures with different polymer ratio, polyelectrolyte deposition, and PC type (mean ± SD, *n* = 3).

PC type and the sequence of polyelectrolytes mixing affected the intensity of PECs precipitation and the morphology of the particles formed (Figure 8). For mixtures with PC 010, a more intense interpolymer complexation was recorded when the polyanion was added first. The highest values of turbidity were noted for 1:2 and 1:1 CS:PC 010 weight ratios. When a different order of polymer deposition was applied, the strongest precipitation was recorded for 1:5 and 1:2 ratios. The use of PC 020 gave the opposite outcomes. The addition of PC 020 to CS solution resulted in visually more intense polycomplexes precipitation compared to the reverse preparation technique; nevertheless, those observations were not confirmed by significantly enhanced turbidity values. The different appearance of CS/PC 020 particles/coacervates might be responsible for the misleading effect of the increased fraction of PECs formed. It cannot be excluded that the high sensitivity of PC 020 to di- but also monovalent ions might result in the hampered complexation by CS. By gradually adding PC to the CS solution, more efficient polymeric interactions might have occurred by replacing counterions with amine groups of CS that were in excess. The highest turbidity was recorded for the ratio of CS to PC 020 1:2 with the polycation added in the first place. It might be concluded that external factors, such as the order of polymer mixing or the polymer ratio, influenced the process of CS/PC PECs formation. The pH changes arising from the increasing amount of acidic CS solution resulted in a slight pH decline to the values corresponding to “pure” CS solution (2.3 ± 0.3). Nevertheless, it should be underlined that the turbidity test does not allow for precise quantitative detection of the phase separation since the ability of light scattering highly depends on the morphological features of the dispersed phase [8]. According to this, observed variations in the turbidity might be affected by both the intensity of PECs’ precipitation/coacervation but also structural differences of the polycomplexes formed.

### 2.6. Zeta Potential

The zeta potential is the charge density on the surface of suspended particles and colloids. The values over 60 mV indicate excellent stability of dispersions, those above 30 mV indicate physical stability, and those below 20 mV are characteristic for limited stability. In addition, a zeta potential lower than 5 mV induces an agglomeration process [42]. In the case of PECs, neutralization of the charge highly corresponds to the enhanced aggregation processes and the lowest solubility at this point.

**Figure 9:**
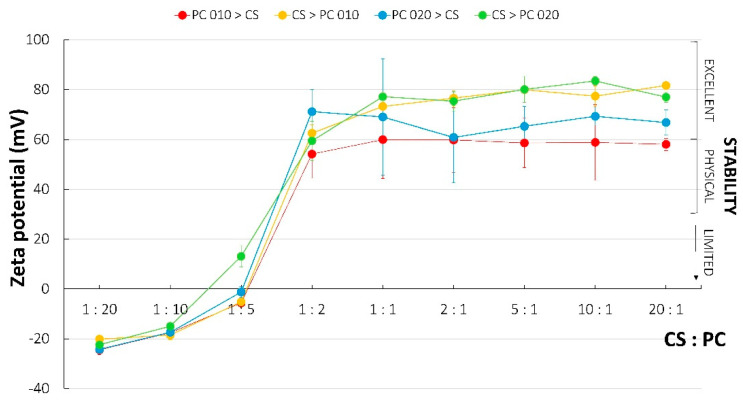
Changes of zeta potential noted for CS/PC mixtures with different polymer ratio and polyelectrolyte deposition. The range of zeta potential being characteristic of excellent, physical, and limited stability was also marked [42] (mean ± SD, *n* = 3).

In this study, changes in the zeta potential values were similar regardless of PC type and the applied order of polyelectrolytes mixing (Figure 9). What was interesting was that CS addition resulted in very high zeta potential, indicating excellent stability of the PECs dispersions [42]. While the point of charge neutralization was observed for the ratio of CS to PC 010 close to 1:5, the addition of CS to PC 020 led to stoichiometric PECs preparation at the point between the 1:10 and 1:5 ratios. Zeta potential values recorded for samples based on PC 020 were inconsistent, since an addition of CS to PC solution resulted in more rapid stoichiometric PEC formation as the turbidity test confirmed. Simultaneously, the range of CS:PC weight ratios 1:10–1:5 was recognized as relevant to charge neutralization.

### 2.7. FTIR Assay

“Pure” polyelectrolytes, films, and corresponding physical mixtures were assessed by FTIR (Figure S1). As previously observed [24], spectra of the physical mixtures were dominated by signals coming from PC, especially for the CS/PC 010 combinations. Among bands characteristic of the polyanion, C=O stretching of methylated carboxyl groups (1733 cm^−1^ and 1730 cm^−1^ for PC 010 and PC 020, respectively) and symmetric stretching vibrations of carboxylic groups (1421 cm^−1^ and 1424 cm^−1^ for PC 010 and PC 020, respectively) were recorded. A signal coming from N–H stretching of amine groups was noted as identifying CS at a wavenumber of 1650 cm^−1^. The physical mixtures of CS and PC showed bands characteristic for both components without shifts, which could indicate potential interpolymer interactions. For comparison, shifts of the bands specific for both amine and carboxylic groups observed for all analyzed films might be a result of the polycomplexes formation [21,24,43]. While ten-fold higher PC content resulted in a “well-preserved” PC layer in F5–F6 films, with less affected bands characteristic for the polyanion, dominance of CS in the composites F9–F12 resulted in equally modified surfaces of the films. An exception to those observations was the FTIR spectrum recorded for F8, which revealed a very similar bands distribution for both tested sides.

## 3. Materials and Methods

### 3.1. Materials

Medium-molecular-weight CS 80/50 (degree of deacetylation: 77.6–82.5%, viscosity of 1% solution in 1% acetic acid: 31–70 mPa·s, molecular weight: 80–200 kDa) was obtained from Heppe Medical CS GmbH (Haale, Germany). Low-methoxy amidated PC with low- (a type CF 010, degree of esterification 34%, degree of amidation 17%, pH 4.2 for 2.5% solution in distilled water at 20 °C) and high-calcium reactivity (a type CF 020, degree of esterification 31%, degree of amidation 19%, pH 4.1 for 2.5% solution in distilled water at 20 °C) were kindly gifted by Herbstreith & Fox and GmbH & Co. KG (Neuenbürg, Germany). Potassium dihydrogen phosphate, sodium chloride, calcium chloride dihydrate, and 85% lactic acid were purchased from Chempur (Piekary Śląskie, Poland). Glycerol was obtained from Fagron (Kraków, Poland).

SSS composed of potassium dihydrogen phosphate (1.63 mg/mL), sodium chloride (2.32 mg/mL), and calcium chloride dihydrate (0.22 mg/mL) with pH 6.2 adjusted by disodium hydrogen phosphate addition (according to Nair et al. with modification [25]) was used for the research analyses.

### 3.2. Methods

#### 3.2.1. Preparation of the Films

**Figure 10:**
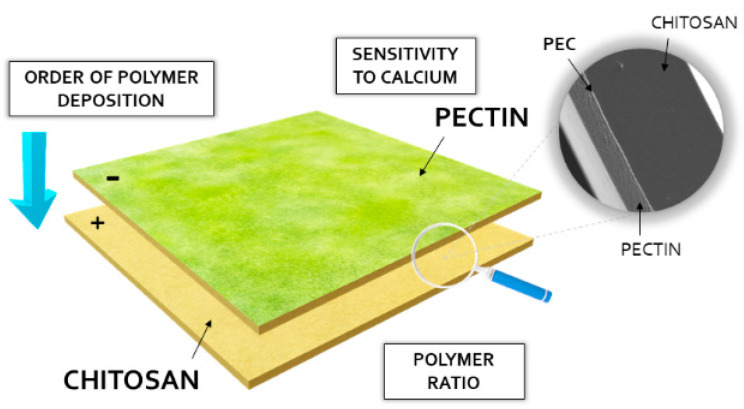
The scheme of the film preparation technique with an indication of the factors being optimized.

Initially, twelve formulations (F1–F12) based on medium-molecular-weight CS and LM PC were prepared. As illustrated in Figure 10, the followed differentiating factors were applied: calcium reactivity of PC, polymer ratio, and order of polyelectrolyte deposition. PCs with low- (PC 010) and high-calcium reactivity (PC 020) but with an identical amount of free carboxyl groups were selected among the variety of PC types available on the market. The selection of a PC that is sensitive to calcium ions resulted from the huge potential of the polymer in the development of smart drug delivery carriers. Three different polymer ratios of CS to PC (10:1, 1:1, and 1:10, *w*/*w*) were assessed. For this purpose, 0.2% and 2% solutions of the polyelectrolytes, with an addition of 0.5% (*w*/*w*) glycerol as both plasticizer and chain interpenetration supporting agent, were utilized. CS solutions were prepared by gradual polymer addition to the mixture of 1% (*w*/*w*) lactic acid and glycerol heated to about 40 °C. PC solutions, as dispersions of the polyelectrolyte in water with the plasticizer, were manufactured at room temperature by using a magnetic stirrer. Since many scientific reports devoted to PECs pointed out the importance of the polymer mixing order concerning the polycomplexes’ characteristics, varied deposition of CS/PC layers was applied (Table 2). To eliminate potential disparities in the performance of the films being a result of a different polymer content, additional F13–F16 films with the polymer ratio 1:1 (*w*/*w*) corresponding to the ratio in F1–F4 and with total polymer content identical to F5–F12 were developed (Table 2). This step was crucial for critical evaluation of the films concerning the impact of interpolymer complexation on the composites’ physicomechanical behavior. The multilayers were prepared according to the previously optimized method [24], then wrapped with aluminum foil and stored at room temperature. Then, 15 g of an adequate polymer solution was placed on a Petri dish in each step of polyelectrolyte deposition by using an analytical balance. Then, they were stored in a fridge to remove air bubbles generated during the mixing processes of the polymer solutions. Films were subsequently dried in the oven at 35–40 °C for about 24 h for pre-gelation of the casted polymer layer.

#### 3.2.2. Evaluation of Mechanical Properties

The 1 × 3 cm film samples were analyzed with regard to their mechanical performance by using Texture Analyzer TA.XT. Plus (Stable Microsystems, Godalming, UK). Mechanical parameters, including σ_s_, ε_s_, E, and TR, were calculated for each of the analyzed composites at room temperature. Test parameters were set based on the previous scientific reports [24,25] and preliminary studies according to the tension test mode. The tensile grips were extended with pre-test, test, and post-test speed of 1 mm/s, with a cell loading of 5 kg, and the initial distance of the grips was 20 mm.

#### 3.2.3. SEM Analysis

Cross-sections and average thickness of the films were evaluated with a scanning electron microscope (In-spect™S50, FEI Company, Hillsboro, OR, USA) at room temperature since the internal structure of the composites was regarded as the most important for the multilayers assessment. Film samples were placed on adhesive tapes fixed to the surface of a special stand and gold sprayed. Different magnifications (1000×–5000×) were utilized.

#### 3.2.4. Swelling and Disintegration Test

The swelling behavior of the films in SSS and purified water was evaluated according to a previously developed and optimized method [23,24,25]. Briefly, 1 cm^2^ films were put in the baskets dedicated for USP dissolution tests [44] and placed in 25 mL beakers with the swelling medium. The beakers were protected with aluminum foil and thermostated in a water bath at 37.0 ± 0.5 °C. At the set time intervals (15, 30, 60, 90, and 120 min), the baskets were removed from the beakers, carefully drained with cellulose wadding, and then weighted using the analytical balance. Mass fluctuations were expressed with the degree of swelling (α), according to the following equation:

α (%) = (W_s_ − W_0_)/W_0_ × 100,

where W_s_—weight of a film after swelling, W_0_—initial weight of a film [45].

#### 3.2.5. Thermal Analysis

Thermogravimetric analyses (TGA) and differential scanning calorimetry analyses (DSC) were performed with a Mettler Toledo Star TGA/DSC unit (Columbus, OH, USA). For TGA analysis, 3–5 mg samples of “pure” CS and PC or their physical mixtures in the weight ratios of 1:10, 1:1, and 10:1 (F20–F25) were placed in aluminum oxide crucibles and heated from 50 °C to 900 °C at a heating rate of 10 °C/min under argon. An empty pan was used as the reference. For the DSC test, 3–5 mg samples of CS, PC, their mixtures (F20–F25), and the films F1–F16 were placed in aluminum crucibles and heated from 0 °C to 480 °C at a heating rate of 10 °C/min under argon, and an empty pan was used as the reference here as well.

#### 3.2.6. Turbidity Test

Turbidity measurements of CS/PC mixtures were performed by using a Hach Model 2100 N IS^®^ Laboratory Turbidimeter (Loveland, CO, USA). Results were presented in nephelometric turbidity units (NTU), signifying the amount of scattered light reaching the detector. To prepare the PEC mixtures, 0.2% (*w*/*w*) CS in 1% (*w*/*w*) lactic acid and 0.2% (*w*/*w*) PC in water with addition of 0.5% (*w*/*w*) glycerol were utilized. With reference to the composition and the preparation technique, different polymer ratios and orders of polymer mixing were applied. Samples with the precipitated PECs were subsequently homogenized at 5000 rpm for 1 min to unify the particle size, then transferred to the sample cell for measurements. All results were performed within 15 min of PEC formation [8,23,46,47]. To recognize the potential impact of pH on the PEC formation process, the pH of the obtained dispersions was also recorded [24].

#### 3.2.7. Measurements of Zeta Potential

Zeta potential values of CS/PC mixtures with different polymer ratio, PC type, and order of polymer mixing were determined with Zetasizer NanoZS90 (Malvern Instruments, Malvern, UK) [42].

#### 3.2.8. FTIR Analysis

The attenuated total reflection FTIR (ATR–FTIR) spectra were made for single polyelectrolytes, physical mixtures, and both sides of films F1–F16. Spectra were recorded using Thermo Scientific Nicolet 6700 FTIR spectrophotometer (Thermo Scientific, Madison, WI, USA) equipped with an ATR accessory. Spectra were recorded against the background spectra and collected in the wavenumber range from 4000 cm^−1^ to 500 cm^−1^ by combining 32 scans with a resolution of 4 cm^−1^.

#### 3.2.9. Statistical Analysis

The quantitative variables were expressed as the mean ± SD using MS Excel software. The measurements of mechanical properties were considered significant at *p* < 0.05.

## 4. Conclusions

Multilayer films consisting of medium-molecular-weight CS and LM PC were developed and assessed as potential buccal drug delivery materials. Different parameters, including PC type, polymer ratio, and deposition order, were considered in the optimization process. Analysis of the mechanical properties and visual evaluation of the films enabled the selection of the most promising composites F1–F4, characterized by homogeneous appearance and optimal mechanical strength for maintaining physical integrity upon 2 h swelling. As SEM images illustrated, a layered structure of the systems was obtained. The CS/LM PC PECs were characterized by improved physicomechanical and thermal stability, and the ionic character of interpolymer bonds was confirmed inter alia by the FTIR analysis. LM PC was able to preserve its unique swelling behavior upon contact with calcium ions; nevertheless, the intensity of polymer–polymer interactions noticeably influenced the capability of water uptake. Considering the complex and multicompartment character of the highest-rated films, their potential in manufacturing buccal films cannot be ignored. The performed optimization was regarded as key for further investigations of multilayer films as antimicrobial agent carriers.

## Figures and Tables

**Table 1:** Mechanical properties of the films F1–F16 expressed as tensile strength (σ_s_) and tear resistance (TR) (mean ± SD, *n* ≥ 3, *p* < 0.05).

Formulation	σ_s_ (N/mm^2^)	TR (N)
F1	28.2 ± 0.0	27.2 ± 2.7
F2	16.6 ± 7.4	9.6 ± 2.5
F3	22.9 ± 6.1	24.6 ± 12.0
F4	44.4 ± 11.8	28.7 ± 2.9
F5	14.8 ± 3.6	10.0 ± 2.5
F6	13.7 ± 0.2	8.0 ± 0.3
F7	4.3 ± 1.9	4.0 ± 1.4
F8	1.5 ± 1.2	2.9 ± 0.7
F9	9.6 ± 4.3	7.8 ± 4.0
F10	8.4 ± 3.2	6.8 ± 3.5
F11	6.9 ± 1.2	5.7 ± 0.6
F12	6.7 ± 1.8	5.1 ± 1.8
F13	11.1 ± 3.2	6.1 ± 1.9
F14	9.8 ± 2.3	5.0 ± 0.6
F15	15.9 ± 9.5	6.0 ± 3.6
F16	10.0 ± 0.7	6.0 ± 0.4

**Table 2:** Composition of F1–F16 films.

Formulation	CS:PC	I Layer	II Layer
F1 ^1^	1:1	2%CS	2%PC010
F2 ^1^		2%CS	2%PC020
F3 ^1^		2%PC010	2%CS
F4 ^1^		2%PC020	2%CS
F5	1:10	0.2%CS	2%PC010
F6		0.2%CS	2%PC020
F7		2%PC010	0.2%CS
F8		2%PC020	0.2%CS
F9	10:1	2%CS	0.2%PC010
F10		2%CS	0.2%PC020
F11		0.2%PC010	2%CS
F12		0.2%PC020	2%CS
F13 ^1^	1:1	1.1%CS	1.1%PC010
F14 ^1^		1.1%CS	1.1%PC020
F15 ^1^		1.1%PC010	1.1%CS
F16 ^1^		1.1%PC020	1.1%CS

^1^ Formulations with identical polymer ratio but different total polymer content.

## Data Availability

Except for the FTIR measurements available in the Supplementary Materials, data are contained within the article.

## Supplementary Materials

The following supporting information can be downloaded at: https://www.mdpi.com/article/10.3390/ijms23158092/s1.
