# Supplementary material for: Optimization of Multilayer Films Composed of Chitosan and Low-Methoxy Amidated Pectin as Multifunctional Biomaterials for Drug Delivery
Source: Int J Mol Sci. 2022 Jul 22;23(15):8092. doi: 10.3390/ijms23158092 (PMC9331940; doi:10.3390/ijms23158092)
Supplement: Supplementary file 1 [file ijms-23-08092-s001.zip › ijms-1803271-supplementary.pdf]

Supplementary Material

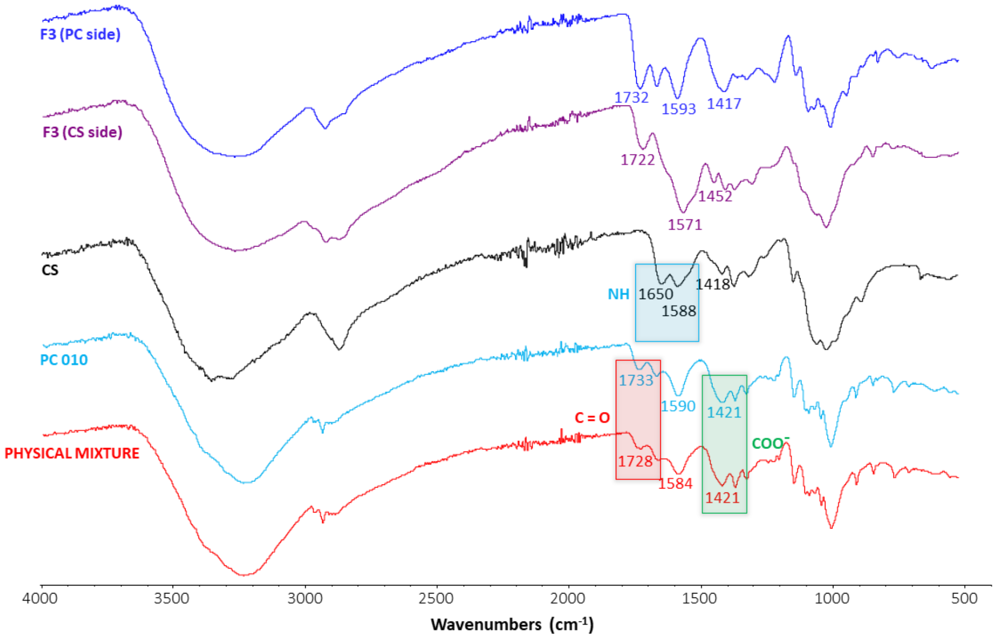

Figure S1. Representative FTIR spectra of chitosan (CS), pectin 010 (PC 010), F3, and the corresponding physical mixture (CS:PC 010 1:1).
